# Human Genetic Disorders Resulting in Systemic Selenoprotein Deficiency

**DOI:** 10.3390/ijms222312927

**Published:** 2021-11-29

**Authors:** Erik Schoenmakers, Krishna Chatterjee

**Affiliations:** Metabolic Research Laboratories, Wellcome Trust-MRC Institute of Metabolic Science, Addenbrooke’s Hospital, University of Cambridge, Cambridge CB2 0QQ, UK; es308@cam.ac.uk

**Keywords:** selenoprotein deficiency, SECISBP2, Sec-tRNA^[Ser]Sec^, SEPSECS, selenium

## Abstract

Selenium, a trace element fundamental to human health, is incorporated as the amino acid selenocysteine (Sec) into more than 25 proteins, referred to as selenoproteins. Human mutations in *SECISBP2*, *SEPSECS* and *TRU-TCA1-1*, three genes essential in the selenocysteine incorporation pathway, affect the expression of most if not all selenoproteins. Systemic selenoprotein deficiency results in a complex, multifactorial disorder, reflecting loss of selenoprotein function in specific tissues and/or long-term impaired selenoenzyme-mediated defence against oxidative and endoplasmic reticulum stress. *SEPSECS* mutations are associated with a predominantly neurological phenotype with progressive cerebello-cerebral atrophy. Selenoprotein deficiency due to *SECISBP2* and *TRU-TCA1-1* defects are characterized by abnormal circulating thyroid hormones due to lack of Sec-containing deiodinases, low serum selenium levels (low SELENOP, GPX3), with additional features (myopathy due to low SELENON; photosensitivity, hearing loss, increased adipose mass and function due to reduced antioxidant and endoplasmic reticulum stress defence) in *SECISBP2* cases. Antioxidant therapy ameliorates oxidative damage in cells and tissues of patients, but its longer term benefits remain undefined. Ongoing surveillance of patients enables ascertainment of additional phenotypes which may provide further insights into the role of selenoproteins in human biological processes.

## 1. Introduction

**Figure 1:**
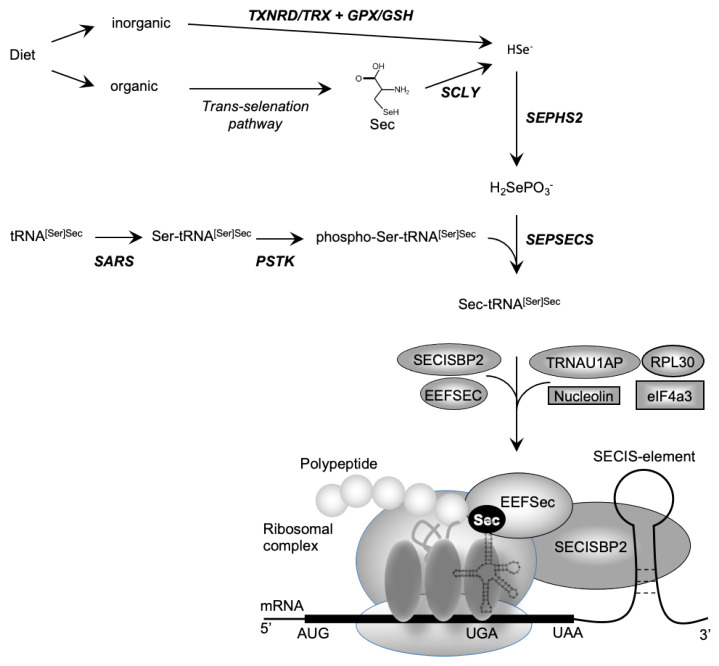
Biosynthesis of selenocysteine (Sec) and selenoproteins. Dietary sources of selenium exist in inorganic form (e.g., selenate, selenite) and organic form (e.g., Sec, SeMet). Inorganic selenium is reduced to selenide by TXNRD/TRX or GPX/GSH systems and organic selenium is metabolized to Sec, used by SCLY to generate selenide. De novo Sec synthesis takes place on its own tRNA (tRNA^[Ser]Sec^), which undergoes maturation through sequential modifications (SARS-mediated addition of Ser, PSTK-mediated phosphorylation of Ser), with acceptance of a selenophosphate (generated from selenide by SEPHS2) catalysed by SEPSECS as final step. Expression of selenoproteins requires recoding of an UGA codon as the amino acid Sec instead of a premature stop. The incorporation of Sec is mediated by a multiprotein complex containing SECISBP2, bound to the SECIS element situated in the 3′-untranslated region of selenoproteins, the Sec elongation factor EEFSEC, together with Sec-tRNA^[Ser]Sec^ at the ribosomal acceptor site. The other factors (e.g., ribosomal protein L30, eukaryotic initiation factor eIF4a3, nucleolin) have regulatory roles.

Dietary selenium (Se) is absorbed as inorganic Se (e.g., selenate; selenite) or organic Se (e.g., Se-methionine; selenocysteine) and metabolized to hydrogen selenide (H_2_Se) before incorporation into the amino acid selenocysteine (Sec) [1]. Selenocysteine is different from other amino acids in that, uniquely, it is synthesized on its own tRNA, (Sec-tRNA^[Ser]Sec^; encoded by *TRU-TCA1-1*), via a well described pathway including O-phosphoserine-tRNA:selenocysteine tRNA synthase (SEPSECS) (Figure 1) [2,3]. Selenocysteine is incorporated into selenoproteins, at the position of a UGA codon in the mRNA, which ordinarily encodes a termination codon that dictates the cessation of protein synthesis. Unique Sec-insertion machinery, involving a cis-acting SEleniumCysteine Insertion Sequence (SECIS) stem-loop structure located in the 3′-UTR of all selenoprotein mRNAs and the UGA codon, interacting with trans-acting factors (SECIS binding protein 2 (SECISBP2), Sec-tRNA specific eukaryotic elongation factor (EEFSEC) and Sec-tRNA^[Ser]Sec^) (Figure 1), recodes UGA as a codon mediating Sec incorporation rather than termination of protein translation [3,4,5].

At least 25 human selenoproteins are described and recognized functions include maintenance of redox potential, regulating redox sensitive biochemical pathways, protection of genetic material, proteins and membranes from oxidative damage, metabolism of thyroid hormones, regulation of gene expression and control of protein folding (Table 1) [3,6]. The importance of selenoproteins is illustrated by the embryonic lethal phenotype of *Trsp* (mouse Sec-tRNA^[Ser]Sec^) and *Secisbp2* knockout mice [7,8]. It is well known that dietary Se intake affects systemic Se-status and selenoprotein expression, but not all selenoproteins are affected to the same extent. Thus, expression of housekeeping selenoproteins (e.g., TXNRD1, TXNRD3, GPX4) is less affected by low circulating Se-levels compared to stress-related selenoproteins (e.g., GPX1, GPX3, SELENOW). Such differential preservation of selenoprotein expression is attributed to the existence of a “hierarchy of selenoprotein synthesis”, whose underlying molecular basis is unclear [3,9]. With this knowledge, it is no surprise that mutations in key components of the Sec-insertion pathway (*SEPSECS*, *SECISBP2*, *TRU-TCA1-1*) result in generalized deficiency of selenoproteins associated with a complex, multisystem phenotypes. Here, we describe the clinical consequences of mutations in these three human genes and suggest possible links with loss-of-function of known selenoproteins.

## 2. *SECISBP2* Mutations

**Figure 2:**
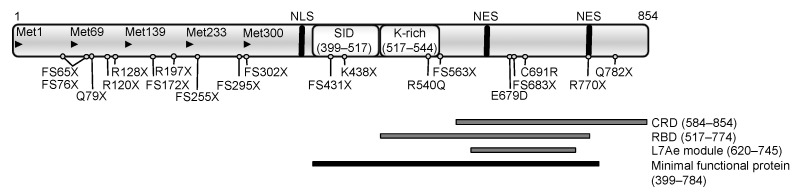
Functional domains of human SECISBP2 with the position of mutations described hitherto. Arrowheads denote the location of ATG codons; NLS: nuclear localisation signal (380–390); NES: nuclear export signals (634–657 and 756–770); SID: Sec incorporation domain; CRD: cysteine rich domain; RBD: minimal RNA-binding domain with the Lysine-rich domain (K-rich) and the L7Ae RNA-binding module; the black bar denotes the minimal protein region required for full functional activity in vitro.

SECISBP2 is an essential and limiting factor for biosynthesis of selenoproteins [4,10] and functions as a scaffold, recruiting ribosomes, EEFSEC, and Sec-tRNA^[Ser]Sec^ to the UGA codon by binding to SECIS-elements in selenoprotein mRNAs, generating a dynamic ribosome-Sec-incorporation complex (Figure 1). The first 400 amino (N-)terminal residues of SECISBP2 have no clear function; in contrast the carboxy (C-)terminal region (amino acids 399–784) is both necessary and sufficient for Sec-incorporation (Sec incorporation domain: SID) and binding to the SECIS element (RNA-binding domain: RBD) in vitro (Figure 2). The RBD, contains a L7Ae-type RNA interaction module and a lysine-rich domain, mediating specific recognition of ‘‘stem-loop’’ structures adopted by SECIS elements and other regulatory RNA motifs [11,12,13]. The C-terminal region also contains motifs (nuclear localization signal; nuclear export signal) involved in cellular localization of SECISBP2 and a cysteine rich domain (Figure 2) [14]. In the N-terminal region, alternative splicing events and ATG start codons have been described, generating different *SECISBP2* isoforms [14], but all containing the essential C-terminal region. These events, together with regulatory domains in the C-terminal region, are thought to control SECISBP2-dependent Sec incorporation and the hierarchy of selenoprotein expression in vivo. 

Homozygous or compound heterozygous mutations in *SECISBP2* have been described in individuals from 11 families from diverse ethnic backgrounds [15] (Table 2, Figure 2); hitherto no phenotypes have been described in heterozygous individuals. Most *SECISBP2* mutations identified to date result in premature stops in the N-terminal region upstream of an alternative start codon (Met 300), permitting synthesis of the shorter, C-terminal, minimal functional domain of SECISBP2 (Figure 2) [14,16,17,18,19,20,21,22,23]. Conversely, stop mutations (e.g., R770X, Q782X) [18,23], distal to the minimal functional domain might generate C-terminally truncated proteins with residual but altered function. In one patient with an intronic mutation (IVS8ds + 29G > A) leading to a stop in the SID-domain, correct mRNA splicing was only reduced by 50% [16], a mechanism preserving some SECISBP2 synthesis that may operate in other splice site mutation contexts.

Only three missense *SECISBP2* mutations, situated in the RBD (R540Q, E679D and C691R) are described. The R540Q mutation, in the lysine-rich domain, fails to bind a specific subset of SECIS-elements in vitro and a mouse model revealed an abnormal pattern of Secisbp2 and selenoprotein expression in tissues [16,24,25]. The E679D and C691R mutations are situated in the L7Ae RNA-binding module and part of the CRD. C691R mutant SECISBP2 undergoes enhanced proteasomal degradation, with loss of RNA-binding [19,25]. The E679D mutation has not been investigated but is predicted to be deleterious (PolyPhen-2 algorithm score of 0.998), possibly affecting RNA-binding [23]. 

Complete knockout of *Secisbp2* in mice is embryonic lethal [8], suggesting some functional protein, or an alternative rescue mechanism, is present in humans with *SECISBP2* mutations. Studies suggest that most combinations of *SECISBP2* mutations in patients hitherto are hypomorphic, with at least one allele directing synthesis of protein at either reduced levels or that is partially functional (Table 2). Because it is rate limiting for Sec incorporation, decreased SECISBP2 function will affect most if not all selenoprotein synthesis, as confirmed by available selenoprotein expression data in the patients [16,19].

Hitherto, only a small number of patients are described, from different ethnic and geographical backgrounds, often with compound heterozygous mutations and with limited information of their phenotypes. Some clinical phenotypes are attributable to deficiencies of particular selenoproteins in specific tissues whilst other features have a complex, multifactorial, basis possible linked to unbalanced antioxidant defence or protein folding pathways or loss of selenoproteins of unknown function. Increased cellular oxidative stress, readily measurable in most cells and tissues from patients, results in cumulative membrane and DNA damage. A common biochemical signature in all patients consists of low circulating selenium (reflecting low plasma SELENOP and GPX3) and abnormal thyroid hormone levels due to diminished activity of deiodinases resulting in raised FT4, normal to low FT3, raised reverse T3 and normal or high TSH concentrations [15,16,19]. Most cases were diagnosed in childhood with growth retardation (e.g., failure to thrive, short stature, delayed bone age) and developmental delay (e.g., delayed speech, intellectual- and motor coordination deficits) as common features, due not only to abnormal thyroid hormone metabolism [26,27] but also effects of specific selenoproteins deficiency in tissues (e.g., neuronal [8] or skeletal [28]). Fatigue and muscle weakness is a recognized feature in several patients and is attributable at least in part to a progressive muscular dystrophy affecting axial and proximal limb muscles, and very similar to the phenotype of myopathy due to selenoprotein N-deficiency [29]. Mild, bilateral, high-frequency sensorineural hearing loss is observed in some patients and is possibly due to ROS-mediated damage in the auditory system [30,31] as it is progressive in nature, worsening in older patients. An adult male patient was azoospermic, with marked deficiency of testis-expressed selenoproteins (GPX4, TXNRD3, SELENOV) [32,33,34,35,36]. Several other recorded phenotypes (increased whole body, subcutaneous fat mass, increased systemic insulin sensitivity, cutaneous photosensitivity) probably have a multifactorial basis which includes loss of antioxidant and endoplasmic reticulum stress defence. Studies of mouse models and in humans provide a substantial body of evidence to suggest a link between selenoproteins and most of these phenotypes [19,37,38,39,40,41].

Clinical management of these patients is mostly limited to correcting abnormal thyroid hormone metabolism with liothyronine supplementation if necessary. No specific therapies exist for other phenotypes (e.g., myopathy), but their progressive nature can require supportive intervention (e.g., nocturnal assisted ventilation for respiratory muscle weakness, aid for hearing loss). Oral selenium supplementation did raise total serum Se levels in some SECISBP2-deficient patients, but without clinical [17,18,21] or biochemical (circulating GPX’s, SELENOP, thyroid hormone metabolism) effect [42]. Antioxidant (alpha tocopherol) treatment was beneficial in one patient, reducing circulating levels of products of lipid peroxidation with reversal of these changes after treatment withdrawal [40]. These observations suggest that treatment with antioxidants is a rational therapeutic approach, but the longterm consequences in this multisystem disorder are unpredictable.

## 3. *TRU-TCA1-1* Mutations

**Figure 3:**
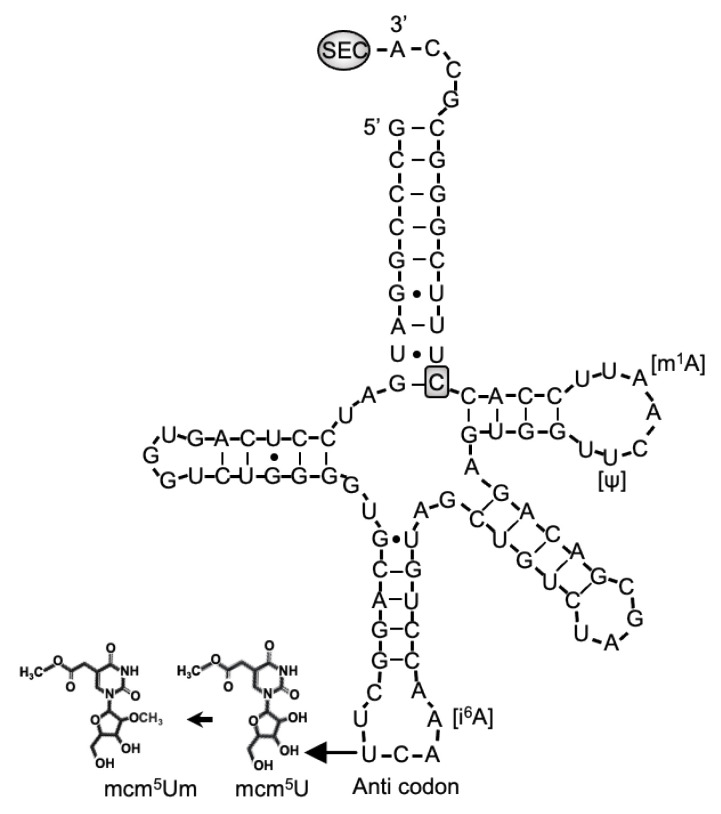
Sec-tRNA^[Ser]Sec^ showing the position of human mutation. The primary structure of human Sec-tRNA^[Ser]Sec^ is shown in a cloverleaf model, with the location of C65G mutation and posttranscriptional modification at positions U34 (mcm^5^U or mcm^5^Um, in the anticodon), A37 (i^6^A), U55 (⍦) and A58 (m^1^A).

Selenocysteine is different from other amino acids in that it is synthesized uniquely on its own tRNA, encoded by *TRU-TCA1-1*, via a well described pathway including SEPSECS (Figure 1) [2,3]. Two major isoforms of the mature Sec-tRNA^[Ser]Sec^ have been identified, containing either 5-methoxycarbonyl-methyluridine (mcm^5^U) or its methylated form 5-methoxycarbonylmethyl-2′-O-methyluridine (mcm^5^Um) at position 34, with the level of methylation being dependent on selenium status (Figure 3). The methylation state of uridine 34, located in the anticodon loop, is thought to contribute to stabilization of the codon–anticodon interaction and to play a role in mediating the hierarchy of selenoprotein expression. Thus, expression of essential, cellular housekeeping selenoproteins (e.g., TXNRDs, GPX4) is dependent on the mcm^5^U isoform, whilst synthesis of cellular, stress-related selenoproteins (e.g., GPX1, GPX3) synthesis require the mcm^5^Um isoform [43,44].

The first patient with a homozygous nucleotide change at position 65 (C > G) in *TRU-TCA1-1* (Figure 3) [45], presented with a similar clinical and biochemical phenotype (fatigue and muscle weakness, raised FT4, normal T3, raised rT3 and TSH, low plasma selenium concentrations) to that seen in patients with *SECISBP2* deficiency. However, the pattern of selenoprotein expression in his cells differed, with preservation housekeeping selenoproteins (e.g., TXNRDs, GPX4), but not stress-related selenoproteins (e.g., GPX1, GPX3) in cells from the *TRU-TCA1-1* mutation patient. This pattern is similar to the differential preservation of selenoprotein synthesis described in murine Sec-tRNA^[Ser]Sec^ mutant models [3,44]. Recently, a second, unrelated patient with the same, homozygous *TRU-TCA1-1* mutation (C65G) with raised FT4 and low plasma GPX3 levels has been described [46].

The mechanism for such differential selenoprotein expression is unresolved, but a possible explanation is the observation that the *TRU-TCA1-1* C65G mutation results in lower total Sec-tRNA^[Ser]Sec^ expression in patients cells, with disproportionately greater diminution in Sec-tRNA^[Ser]Sec^ mcm^5^Um levels. This suggest that the low Sec-tRNA^[Ser]Sec^ levels in the proband are still sufficient for normal synthesis of housekeeping selenoproteins, whereas diminution of Sec-tRNA^[Ser]Sec^ mcm^5^Um levels accounts for reduced synthesis of stress-related selenoproteins.

Clinical management of the patient is limited to alleviating clinical symptoms. However, with the knowledge that changing systemic selenium status can alter the relative proportions of the Sec-RNA^[Ser]Sec^ isoforms [44,47,48], selenium supplementation of this patient, aiming to restore specific selenoprotein deficiencies, may be a rational therapeutic approach.

## 4. *SEPSECS* Mutations

The human SEPSECS protein was first characterized as an autoantigen (soluble liver antigen/liver pancreas, SLA) in autoimmune hepatitis [49]. The observation that it was present in a ribonucleoprotein complex with Sec-tRNA^[Ser]Sec^, led to its identification as the enzyme that catalyzes the final step of Sec formation by converting O-phosphoserine-tRNA^[Ser]Sec^ into Sec-tRNA^[Ser]Sec^ using selenophosphate as substrate donor [50,51] (Figure 1).

**Figure 4:**
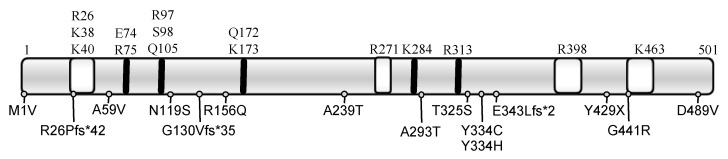
Functional domains of SEPSECS with the positions of the human mutations. Schematic of the human SEPSECS protein with key amino acids (above) that are part of the active domain (black bars) or interact with tRNA^[ser]sec]^ (white shaded boxes) and mutations described hitherto below.

Homozygous and compound heterozygous mutations in *SEPSECS* have been identified in 20 patients (Table 3, Figure 4). The availability of the crystal structures of the archaeal and murine SEPSECS apo-enzymes as well as human wild type and mutant SECSEPS (A239T, Y334C, T325S and Y429X) complexed with Sec-tRNA^[Ser]Sec^ provides functional information [52,53,54,55]. The four premature stop mutants are predicted to be insoluble and inactive, as documented for the Y429X mutant. Mutants at Tyrosine 334 are predicted to fold like wild type SEPSECS and retain binding to Sec-tRNA^[Ser]Sec^, but with reduced enzyme activity. The A239T mutant failed to form stable tetramers, possible as result of a steric clash destabilizing the enzyme’s core, rendering it inactive [55]. The other mutants for which no structure is available have been analyzed in silico and are predicted to be deleterious to varying degrees [15].

Patients with mutations in *SEPSECS* have profound intellectual disability, global developmental delay, spasticity, epilepsy, axonal neuropathy, optic atrophy and hypotonia with progressive microcephaly due to cortical and cerebellar atrophy [56,58,61,63]. The disorder is classified as autosomal recessive pontocerebellar hypoplasia type 2D (PCH2D, OMIM#613811), also referred to as progressive cerebellocerebral atrophy (PCCA) [56,67]. SEPSECS is required for generation of Sec-tRNA^[Ser]Sec^, which is essential for survival as demonstrated by the *Trsp* (mouse tRNA^[Ser]Sec^) knockout mouse model [44]. The Y334C-*Sepsecs* mouse model exhibits a phenotype similar to features described in patients [68]. However, there is some variation in impact of *SEPSECS* mutations and specific phenotypes, with three patients (homozygous for G441R; compound heterozygous for R26Pfs*42/N119S or N119S/R156Q), presenting with late-onset PCH2D and progressive but milder degree of CNS atrophy [62,64]. In silico analyses suggest that these mutations have a less deleterious effect on SEPSECS function [15], although environmental factors or patients’ genetic background modulating phenotype cannot be excluded.

The young age and severity of neurological problems in *SEPSECS* patients has precluded detailed investigation of selenoprotein expression and associated phenotypes. Studies of brain tissue from some patients showed decreased selenoprotein expression, correlating with increased cellular oxidative stress, but selenoprotein expression in other cell types (fibroblasts, muscle cells) was not significantly affected [58]. Serum selenium concentrations and thyroid status has been partially investigated in five patients, documenting either normal levels [61,65] or normal T4 but elevated TSH levels [58]. This suggests that the biochemical hallmarks of selenoprotein deficiency in *SECISBP2* and *TRU-TCA1-1* disorders (low circulating selenium and abnormal thyroid hormone levels) are not a significant feature in patients with *SEPSECS* mutations. Myopathic features with raised CK levels, abnormal mitochondria, cytoplasmic bodies and increased lipid accumulation in muscle are documented in one *SEPSECS* mutation case [61], with broad-based gait and postural instability suggesting muscle weakness in another patient [64]. These findings are similar to observations in selenoprotein N-deficient patients with *SECISBP2* mutations [19]. Overall, limited studies to date suggest that *SEPSECS* patients exhibit phenotypes associated with selenoprotein deficiency, but that these features can be mutation and tissue dependent.

## 5. Conclusions

In humans, 25 genes, encoding different selenoproteins containing the amino acid selenocysteine (Sec), have been identified. In selenoprotein mRNAs the amino acid Sec is encoded by the triplet UGA which usually constitutes a stop codon, requiring its recoding by a complex, multiprotein mechanism. Failure of selenoprotein synthesis due to *SECISBP2*, *TRU-TCA1-1* or *SEPSECS* defects, essential components of the selenoprotein biosynthesis pathway, results in complex disorders.

Individuals with *SECISBP2* defects exhibit a multisystem phenotype including growth retardation, fatigue and muscle weakness, sensorineural hearing loss, increased whole body fat mass, azoospermia and cutaneous photosensitivity. Most patients were identified due to a characteristic biochemical signature with raised FT4, normal to low FT3, raised rT3 and normal/slightly high TSH and low plasma selenium levels. A similar biochemical phenotype and clinical features are described in one individual with a *TRU-TCA1-1* mutation, although with relative preservation of essential housekeeping versus stress-related selenoprotein expression in his cells. Individuals with *SEPSECS* defects, essential for Sec-tRNA^[Ser]Sec^ synthesis, present with a disorder characterized by cerebello-cerebral atrophy. Due to the young age and severe phenotype of patients, the effect of *SEPSECS* mutations on selenoprotein expression has not been studied in detail. In contrast, it is noteworthy that an early-onset central nervous system phenotype is not a feature in patients with *SECISBP2* or *TRU-TCA1-1* mutations.

As the function of many selenoproteins is unknown, or simultaneous deficiency of several selenoproteins exerts additive, synergistic or antagonistic effects culminating in complex dysregulation, linking disease phenotypes with altered expression of specific selenoproteins is challenging. Nevertheless, some causal links between specific selenoprotein deficiencies and phenotypes (e.g., abnormal thyroid function and deiodinase enzymes; low plasma Se and SELENOP, GPX3; azoospermia and SELENOV, GPX4, TXRND3; myopathy and SELENON) can be made. Other, progressive, phenotypes (e.g., photosensitivity, age-dependent hearing loss, neurodegeneration) may reflect absence of selenoenzymes mediating defence against oxidative and endoplasmic reticulum stress, resulting in cumulative tissue damage.

Triiodothyronine supplementation can correct abnormal thyroid hormone metabolism, with other medical intervention being mainly supportive. Selenium supplementation is of no proven benefit in *SECISBP2* mutation patients, but needs evaluation in the *TRU-TCA1-1* mutation case. Antioxidants, targeting the imbalance in oxidoredox and protein folding control pathways, could be beneficial in many selenoprotein deficient patients, but due to the complex interplay between different selenoproteins and their role in diverse biological processes, such treatment will require careful evaluation.

## Figures and Tables

**Table 1:** Human selenoproteins.

Selenoprotein	Function	Expression Subcellular Localization
GPX1 glutathione peroxidase 1	Oxidoreductase	most tissues cytoplasmic
GPX2 glutathione peroxidase 2	Oxidoreductase	limited number of tissues Nucleus and cytoplasmic
GPX3 glutathione peroxidase 3	Oxidoreductase	most tissues secreted
GPX4 glutathione peroxidase 4	Oxidoreductase	most tissues Nucleus and mitochondria
GPX6 glutathione peroxidase 6	Oxidoreductase	testis, epididymis, olfactory system predicted secreted
TXNRD1 thioredoxin reductase 1	Oxidoreductase	Ubiquitous Nucleus and cytoplasmic
TXNRD2 Thioredoxin reductase 2	Oxidoreductase	Ubiquitous cytoplasmic and mitochondria
TXNRD3 Thioredoxin reductase 3	Oxidoreductase	most tissues, high in testis Intracellular
DIO1 Iodothyronine deiodinase 1	Thyroid hormone metabolism	kidney, liver, thyroid gland Intracellular membrane-associated
DIO2 Iodothyronine deiodinase 2	Thyroid hormone metabolism	central nervous system, pituitary Intracellular membrane-associated
DIO3 Iodothyronine deiodinase 3	Thyroid hormone metabolism	several tissues Intracellular membrane-associated
MSRB1 methionine sulfoxide reductase B1	Met sulfoxide reduction	Ubiquitous Nucleus and cytoplasmic
SELENOF Selenoprotein F	Protein folding control	Ubiquitous endoplasmic reticulum
SELENOH Selenoprotein H	Unknown oxidoreductase	Ubiquitous Nucleus
SELENOI Selenoprotein I	Phospholipid biosynthesis	Ubiquitous transmembrane
SELENOK Selenoprotein K	Protein folding control	Ubiquitous ER, plasma membrane
SELENOM Selenoprotein M	Unknown	Ubiquitous Nuclear and perinuclear
SELENON Selenoprotein N	Redox-calcium homeostasis	Ubiquitous endoplasmic reticulum
SELENOO Selenoprotein O	Protein AMPylation activity	Ubiquitous mitochondria
SELENOP Selenoprotein P	Transport/oxidoreductase	most tissues secreted, cytoplasmic
SELENOS Selenoprotein S	Protein folding control	Ubiquitous endoplasmic reticulum
SELENOT Selenoprotein T	Unknown oxidoreductase	Ubiquitous endoplasmic reticulum
SELENOV Selenoprotein V	Unknown	thyroid, parathyroid, testis, brain Intracellular
SELENOW Selenoprotein W	Oxidoreductase	Ubiquitous Intracellular
SEPHS2 Selenophosphate synthetase 2	Selenophosphate synthesis	Ubiquitous, high in liver and kidney Intracellular

**Table 2:** Human SECISBP2 mutations.

Age in Years (Gender)	Mutation	Protein Change	Alleles Affected	Ethnicity	Reference
26 (M ^1^); 19 (M); 19 (F ^2^)	c.1619 G > A	R540Q	homozygous	Saudi Arabian	[16]
25 (M)	c.1312 A > T c.IVS8ds + 29 G > A	K438 * fs431 *	compound heterozygous	Irish	[16]
19 (M)	c.382 C > T	R128 *	homozygous	Ghanaian	[17]
18 (F)	c.358 C > T c.2308 C > T	R120 * R770 *	compound heterozygous	Brazilian	[18]
44 (M)	c.668delT c.IVS7 -155, T > A	F223fs255 * fs295 * + fs302 *	compound heterozygous	British	[19]
13 (M)	c. 2017 T > C 1–5 intronic SNP’s	C691R fs65 * + fs76 *	compound heterozygous	British	[19]
15 (M)	c.1529_1541dup CCAGCGCCCCACT c.235 C > T	M515fs563 * Q79 *	compound heterozygous	Japanese	[20]
10 (M)	c.800_801insA	K267Kfs * 2	homozygous	Turkish	[21]
3.5 (M)	c.283delT c.589 C > T	T95Ifs31 * R197 *	compound heterozygous	N/A ^3^	[22]
11 (F)	c.2344 C > T c.2045–2048 delAACA	Q782 * K682fs683 *	compound heterozygous	Turkish	[23]
5 (F)	c.589 C > T c.2108 G > T or C	R197 * E679D	compound heterozygous	Argentinian	[23]

^1^ M: Male; ^2^ F: Female; ^3^ N/A: Not available.

**Table 3:** Human SEPSECS mutations.

Age in Year (Gender)	Mutation	Protein Change	Alleles Affected	Ethnicity	Reference
6 (F ^1^); 7.5 (^2^)	c.1001 A > G	Y334C	homozygous	Jewish/Iraq	[56]
4 (F); 2.5 (M)	c.715 G > A c.1001 A > G	A239T Y334C	compound heterozygous	Iraqi/ Moroccan	[56]
7 (F); 4 (F); 2 (F)	c.1466 A > T	D489V	homozygous	Jordan	[57]
0 (M); 0 (F); 0 (F); 0 (F)	c.974 C > G c.1287 C > A	T325S Y429X	compound heterozygous	Finnish	[58]
14 (F)	c.1 A > G c.388 + 3 A > G	M1V G130Vfs * 5	compound heterozygous	N/A ^3^	[59]
N/A	c.1027–1120del	E343Lfs * 2	Homozygous	N/A ^3^	[60]
9 (M)	c.1001 A > C	Y334H	homozygous	Arabian	[61]
10 (F)	c.77delG c.356 A > G	R26Pfs * 42 N119S	compound heterozygous	Japanese	[62]
21 (F)	c.356 A > G c.467 G > A	N119S R156Q	compound heterozygous	Japanese	[62]
1 (M)	c.176 C > T	A59V	Homozygous	N/A ^3^	[63]
23 (F)	c.1321 G > A	G441R	Homozygous	N/A ^3^	[64]
4 (F)	c.114 + 3 A > G	N/A ^3^	Homozygous	Moroccan	[65]
N/A ^1^	c.877 G > A	A293T	Homozygous	N/A ^3^	[66]

^1^ F: Female; ^2^ M: Male; ^3^ N/A: Not available.

## Data Availability

Not applicable.
